# The genome sequence of the tongue-biting isopod, *Ceratothoa steindachneri *Koelbel, 1878

**DOI:** 10.12688/wellcomeopenres.24289.1

**Published:** 2025-05-27

**Authors:** Chris Fletcher, David Alexander, Bethany Reed, Jessica Thomas, Natural History Museum Genome Acquisition Lab, Darwin Tree of Life Barcoding collective, Wellcome Sanger Institute Tree of Life Management, Samples and Laboratory team, Wellcome Sanger Institute Scientific Operations: Sequencing Operations, Wellcome Sanger Institute Tree of Life Core Informatics team, Tree of Life Core Informatics collective, Darwin Tree of Life Consortium

**Affiliations:** 1 Natural History Museum, London, England, UK; 2 Eco Marine Consultants, Bath, England, UK; 3 Tree of Life, Wellcome Sanger Institute, Hinxton, England, UK

**Keywords:** Ceratothoa steindachneri, tongue-biting isopod, genome sequence, chromosomal, Isopoda

## Abstract

We present a genome assembly from a specimen of *Ceratothoa steindachneri* (tongue-biting isopod; Arthropoda; Malacostraca; Isopoda; Cymothoidae). The genome sequence has a total length of 3,927.82 megabases. Most of the assembly (98.71%) is scaffolded into 23 chromosomal pseudomolecules. The mitochondrial genome has also been assembled, with a length of 29.61 kilobases. Gene annotation of this assembly on Ensembl identified 13,816 protein-coding genes.

## Species taxonomy

Eukaryota; Opisthokonta; Metazoa; Eumetazoa; Bilateria; Protostomia; Ecdysozoa; Panarthropoda; Arthropoda; Mandibulata; Pancrustacea; Crustacea; Multicrustacea; Malacostraca; Eumalacostraca; Peracarida; Isopoda; Flabellifera; Cymothoidae; *Ceratothoa*; *Ceratothoa steindachneri* Koelbel, 1878 (NCBI:txid2922061)

## Background

The tongue-biting isopod, *Ceratothoa steindachneri*, is an ectoparasite in the family Cymothoidae that parasitises marine teleost fish. In the UK, it has predominantly been associated with the lesser weever fish ( *Echiichthys vipera*). Tongue-biters inhabit the buccal cavity of their hosts, where they feed on blood and tissues. After initially attaching to the host’s tongue and severing its blood supply, *C. steindachneri* causes the tongue to atrophy, ultimately functioning as a replacement. Like other cymothoids, it undergoes protandric hermaphroditism, initially maturing as a male before transitioning into a female after host attachment. Adults reach between 15mm and 25mm in size, with an oval body shape and curved, sharp dactyls. *C. steindachneri* can be distinguished from other UK cymothoids by its head, which is immersed in the first pereonite with the base of the antennae expanded and touching each other ( Horton & Baillie, 2019).

The first UK record of *C. steindachneri* is relatively recent, dating to 1996, from Whitsand Bay, Cornwall ( Horton, 2000). However, museum specimens of *E. vipera* from 1983 were also found to be infected with the parasite ( Horton & Okamura, 2002). In 2002, Horton and Okamura determined *C. steindachneri’s* range to be limited to the south-west of Britain, but it now appears to be more widespread along the southern coast of England. It has been hypothesised that climate change may have played a role in the range expansion of this species.

We present a chromosomally complete genome sequence for *Ceratothoa steindachneri* based on an adult specimen collected from the Thames Estuary, London, UK, as part of the Darwin Tree of Life Project. This project is a collaborative effort to sequence all named eukaryotic species in the Atlantic Archipelago of Britain and Ireland ( Blaxter *et al.*, 2022).

## Genome sequence report

### Sequencing data

**Figure 1.**
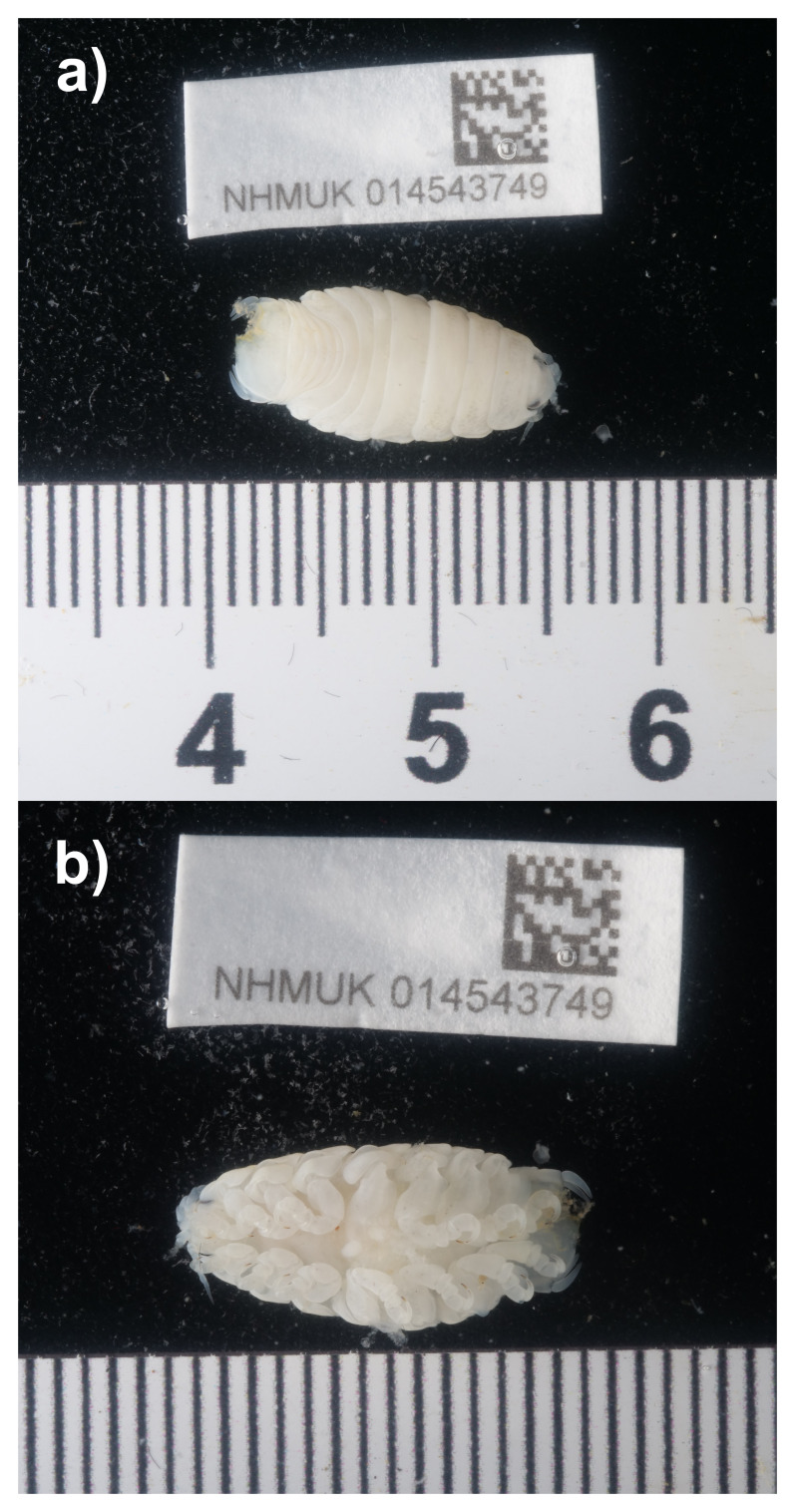
Photographs of the *Ceratothoa steindachneri* (qmCerStei3) specimens used for genome sequencing: **a**) dorsal view, **b**) ventral view.

The genome of a specimen of *Ceratothoa steindachneri* ( Figure 1) was sequenced using Pacific Biosciences single-molecule HiFi long reads, generating 169.40 Gb from 16.56 million reads, which were used to assemble the genome. GenomeScope analysis estimated the haploid genome size at 4,021.58 Mb, with a heterozygosity of 0.22% and repeat content of 36.47%. These estimates guided expectations for the assembly. Based on the estimated genome size, the sequencing data provided approximately 41x coverage. Hi-C sequencing produced 430.72 Gb from 2,852.43 million reads, and was used to scaffold the assembly. RNA sequencing data were also generated and are available in public sequence repositories. Table 1 summarises the specimen and sequencing details.

**Table 1.** Specimen and sequencing data for *Ceratothoa steindachneri*.

Project information			
**Study title**	Ceratothoa steindachneri		
**Umbrella BioProject**	PRJEB72113		
**Species**	*Ceratothoa steindachneri*		
**BioSpecimen**	SAMEA112652167		
**NCBI taxonomy ID**	2922061		
Specimen information			
**Technology**	**ToLID**	**BioSample accession**	**Organism part**
**PacBio long read sequencing**	qmCerStei3	SAMEA112652235	Terminal body
**Hi-C sequencing**	qmCerStei1	SAMEA14448168	Leg
**RNA sequencing**	qmCerStei5	SAMEA112652243	Posterior body
Sequencing information			
**Platform**	**Run accession**	**Read count**	**Base count (Gb)**
**Hi-C Illumina NovaSeq 6000**	ERR12542995	2.85e+09	430.72
**PacBio Revio**	ERR12535454	8.97e+06	93.83
**PacBio Revio**	ERR12535453	7.59e+06	75.58
**RNA Illumina NovaSeq 6000**	ERR12542996	6.68e+07	10.09

### Assembly statistics

The primary haplotype was assembled, and contigs corresponding to an alternate haplotype were also deposited in INSDC databases. The assembly was improved by manual curation, which corrected 157 misjoins or missing joins and removed 4 haplotypic duplications. These interventions decreased the scaffold count by 10.39%. The final assembly has a total length of 3,927.82 Mb in 534 scaffolds, with 2,620 gaps, and a scaffold N50 of 204.26 Mb ( Table 2).

**Table 2.** Genome assembly data for *Ceratothoa steindachneri*.

Genome assembly		
Assembly name	qmCerStei3.1	
Assembly accession	GCA_963969495.1	
*Alternate haplotype accession*	*GCA_963969475.1*	
Assembly level for primary assembly	chromosome	
Span (Mb)	3,927.82	
Number of contigs	3,154	
Number of scaffolds	534	
Longest scaffold (Mb)	256.75	
Assembly metric	Measure	*Benchmark*
Contig N50 length	2.41 Mb	*≥ 1 Mb*
Scaffold N50 length	204.26 Mb	*= chromosome N50*
Consensus quality (QV)	Primary: 56.1; alternate: 56.3; combined: 56.3	*≥ 40*
*k*-mer completeness	Primary: 93.77%; alternate: 90.65%; combined: 98.88%	*≥ 95%*
BUSCO *	C:92.4%[S:91.4%,D:1.0%], F:4.2%,M:3.4%,n:1,013	*S > 90%; D < 5%*
Percentage of assembly assigned to chromosomes	98.71%	*≥ 90%*
Sex chromosomes	Not identified	*localised homologous pairs*
Organelles	Mitochondrial genome: 29.61 kb	*complete single alleles*

*BUSCO scores based on the arthropoda_odb10 BUSCO set using version 5.5.0. C = complete [S = single copy, D = duplicated], F = fragmented, M = missing, n = number of orthologues in comparison.

**Figure 2.**
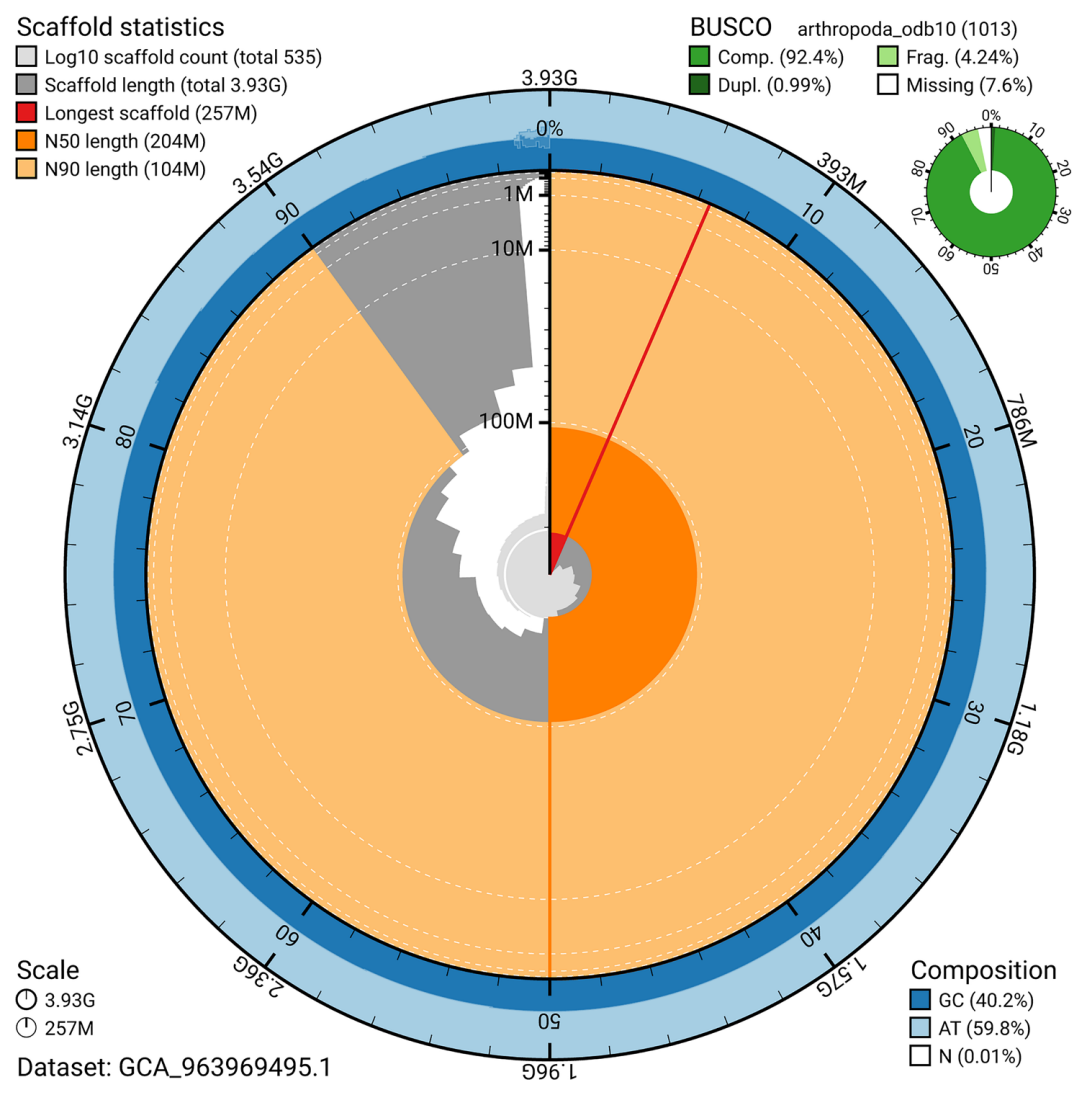
Genome assembly of *Ceratothoa steindachneri*, qmCerStei3.1: metrics. The BlobToolKit snail plot provides an overview of assembly metrics and BUSCO gene completeness. The circumference represents the length of the whole genome sequence, and the main plot is divided into 1,000 bins around the circumference. The outermost blue tracks display the distribution of GC, AT, and N percentages across the bins. Scaffolds are arranged clockwise from longest to shortest and are depicted in dark grey. The longest scaffold is indicated by the red arc, and the deeper orange and pale orange arcs represent the N50 and N90 lengths. A light grey spiral at the centre shows the cumulative scaffold count on a logarithmic scale. A summary of complete, fragmented, duplicated, and missing BUSCO genes in the set is presented at the top right. An interactive version of this figure is available at https://blobtoolkit.genomehubs.org/view/GCA_963969495.1/dataset/GCA_963969495.1/snail.

**Figure 3.**
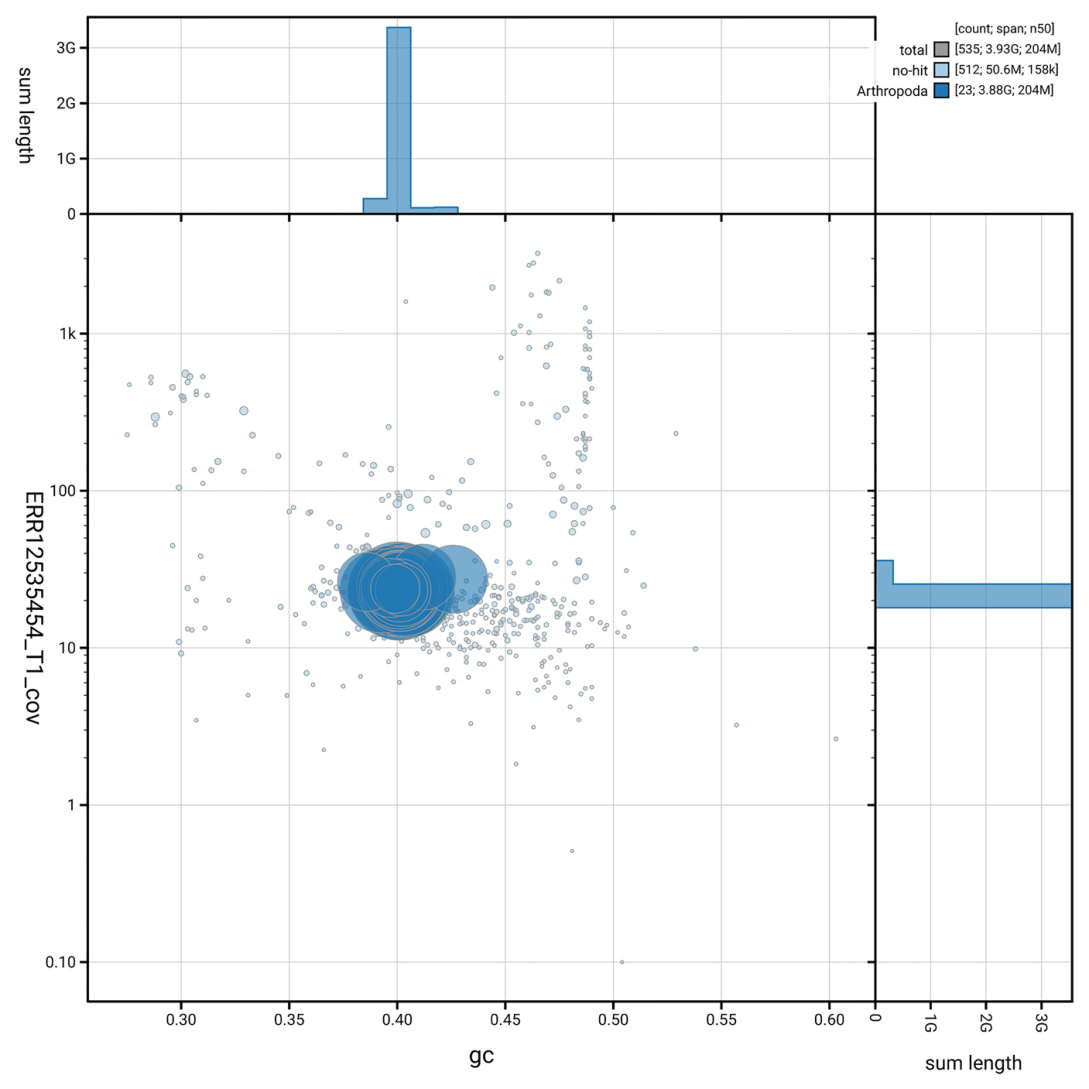
Genome assembly of *Ceratothoa steindachneri*, qmCerStei3.1: BlobToolKit GC-coverage plot. Blob plot showing sequence coverage (vertical axis) and GC content (horizontal axis). The circles represent scaffolds, with the size proportional to scaffold length and the colour representing phylum membership. The histograms along the axes display the total length of sequences distributed across different levels of coverage and GC content. An interactive version of this figure is available at https://blobtoolkit.genomehubs.org/view/GCA_963969495.1/dataset/GCA_963969495.1/blob.

**Figure 4.**
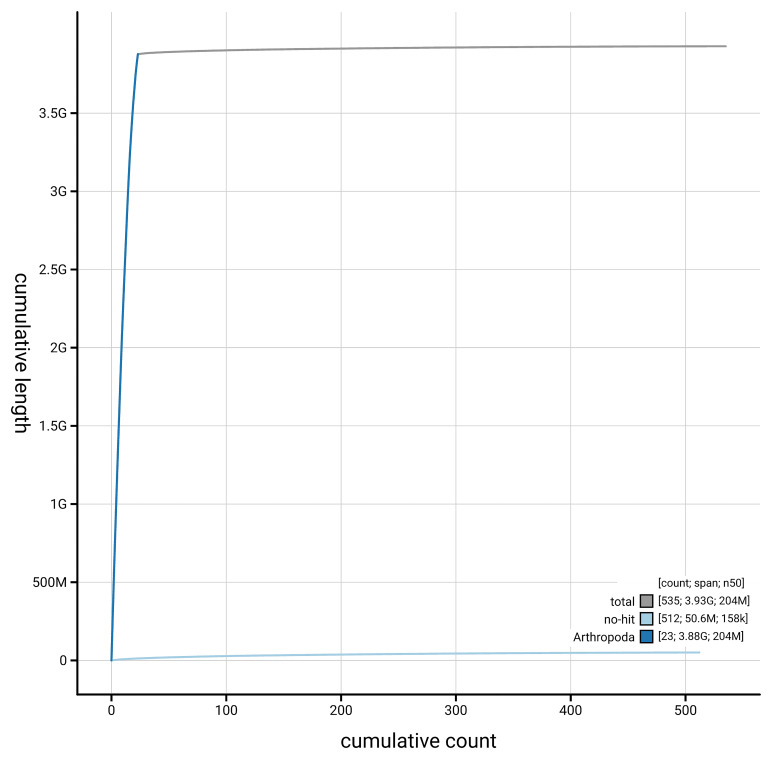
Genome assembly of *Ceratothoa steindachneri,* qmCerStei3.1: BlobToolKit cumulative sequence plot. The grey line shows cumulative length for all scaffolds. Coloured lines show cumulative lengths of scaffolds assigned to each phylum using the buscogenes taxrule. An interactive version of this figure is available at https://blobtoolkit.genomehubs.org/view/GCA_963969495.1/dataset/GCA_963969495.1/cumulative.

The snail plot in Figure 2 provides a summary of the assembly statistics, indicating the distribution of scaffold lengths and other assembly metrics. Figure 3 shows the distribution of scaffolds by GC proportion and coverage. Figure 4 presents a cumulative assembly plot, with separate curves representing different scaffold subsets assigned to various phyla, illustrating the completeness of the assembly.

**Figure 5.**
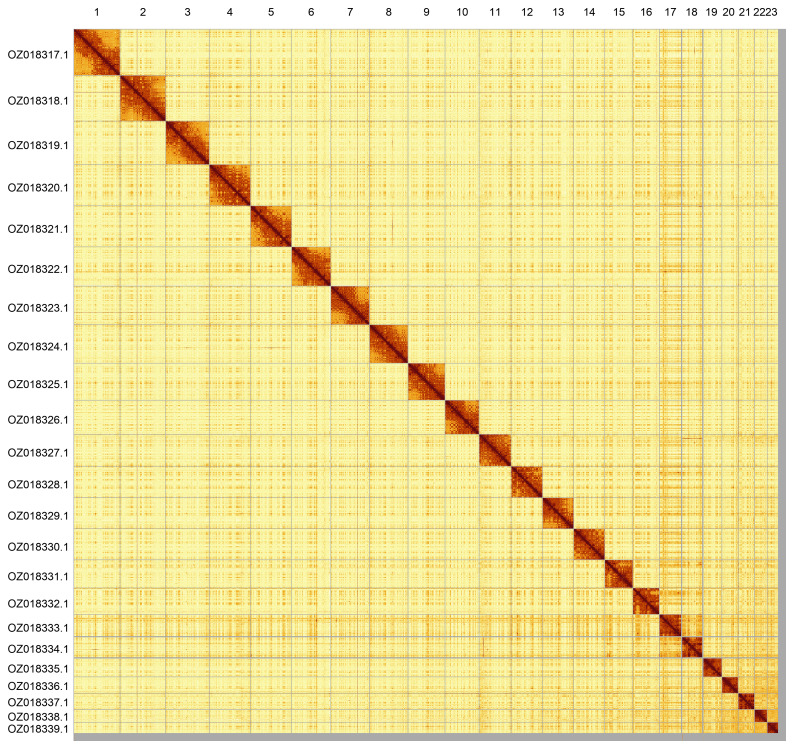
Genome assembly of *Ceratothoa steindachneri*. Hi-C contact map of the qmCerStei3.1 assembly, generated using PretextSnapshot. Chromosomes are shown in order of size and labelled with chromosome numbers (top) and chromosome accession numbers (left).

Most of the assembly sequence (98.71%) was assigned to 23 chromosomal-level scaffolds. These chromosome-level scaffolds, confirmed by Hi-C data, are named according to size ( Figure 5; Table 3). No sex chromosomes were identified during curation.

**Table 3.** Chromosomal pseudomolecules in the genome assembly of *Ceratothoa steindachneri*, qmCerStei3.

INSDC accession	Name	Length (Mb)	GC%
OZ018317.1	1	256.75	40
OZ018318.1	2	252.42	40
OZ018319.1	3	239.4	40
OZ018320.1	4	227.7	40.5
OZ018321.1	5	226.05	40.5
OZ018322.1	6	216.9	40
OZ018323.1	7	212.63	40.5
OZ018324.1	8	212.42	40.5
OZ018325.1	9	204.26	40.5
OZ018326.1	10	187.01	39.5
OZ018327.1	11	177.04	40
OZ018328.1	12	172.89	40
OZ018329.1	13	172.05	40
OZ018330.1	14	171.23	40
OZ018331.1	15	154.96	40
OZ018332.1	16	145.69	40
OZ018333.1	17	121.2	42.5
OZ018334.1	18	112.72	41
OZ018335.1	19	103.74	40
OZ018336.1	20	89.62	39.5
OZ018337.1	21	89.38	38.5
OZ018338.1	22	70.67	40
OZ018339.1	23	60.53	40
OZ018340.1	MT	0.03	46

The mitochondrial genome was also assembled. This sequence is included as a contig in the multifasta file of the genome submission and as a standalone record.

***Assembly quality metrics***

The estimated Quality Value (QV) and *k*-mer completeness metrics, along with BUSCO completeness scores, were calculated for each haplotype and the combined assembly. The QV reflects the base-level accuracy of the assembly, while *k*-mer completeness indicates the proportion of expected *k*-mers identified in the assembly. BUSCO scores provide a measure of completeness based on benchmarking universal single-copy orthologues.

The combined primary and alternate assemblies achieve an estimated QV of 56.3. The *k*-mer recovery for the primary haplotype is 93.77%, and for the alternate haplotype 90.65%; the combined primary and alternate assemblies have a *k*-mer recovery of 98.88%. BUSCO v.5.5.0 analysis using the arthropoda_odb10 reference set ( *n* = 1,013) identified 92.4% of the expected gene set (single = 91.4%, duplicated = 1.0%).

Table 2 provides assembly metric benchmarks adapted from Rhie *et al.* (2021) and the Earth BioGenome Project Report on Assembly Standards September 2024. The assembly achieves the EBP reference standard of **6.C.Q56.**

***Genome annotation report***

The *Ceratothoa steindachneri* genome assembly (GCA_963969495.1) was annotated externally by Ensembl at the European Bioinformatics Institute (EBI). This annotation includes 35,257 transcribed mRNAs from 13,816 protein-coding and 9,044 non-coding genes. The average transcript length is 36,991.74 bp. There are 1.54 coding transcripts per gene and 5.78 exons per transcript. For further information about the annotation, please refer to https://beta.ensembl.org/species/e190dcfd-c668-4a53-a0fa-897229b63dd8.

## Methods

### Sample acquisition and DNA barcoding

The specimen used for genome sequencing was an adult *Ceratothoa steindachneri* (specimen ID NHMUK014543749, ToLID qmCerStei3). A second specimen was used for Hi-C sequencing (specimen ID NHMUK014552859, ToLID qmCerStei1), and a third specimen was used for RNA sequencing (specimen ID NHMUK014543750, ToLID qmCerStei5). All specimens were collected from the Thames Estuary, England, United Kingdom (latitude 51.5, longitude 0.78) on 2022-04-12 by trawl. The specimens were collected by Chris Fletcher (Natural History Museum), David Alexander (Eco Marine Consultants) and Bethany Reed (Eco Marine Consultants), identified by Chris Fletcher, and then preserved dry freezing (–80 °C).

The initial identification was verified by an additional DNA barcoding process according to the framework developed by Twyford *et al*. (2024). A small sample was dissected from the specimen and stored in ethanol, while the remaining parts were shipped on dry ice to the Wellcome Sanger Institute (WSI) ( Pereira *et al.*, 2022). The tissue was lysed, the COI marker region was amplified by PCR, and amplicons were sequenced and compared to the BOLD database, confirming the species identification ( Crowley *et al.*, 2023). Following whole genome sequence generation, the relevant DNA barcode region was also used alongside the initial barcoding data for sample tracking at the WSI ( Twyford *et al.*, 2024). The standard operating procedures for Darwin Tree of Life barcoding have been deposited on protocols.io ( Beasley *et al.*, 2023).

Metadata collection for samples adhered to the Darwin Tree of Life project standards described by Lawniczak *et al*. (2022).

### Nucleic acid extraction

The workflow for high molecular weight (HMW) DNA extraction at the Wellcome Sanger Institute (WSI) Tree of Life Core Laboratory includes a sequence of procedures: sample preparation and homogenisation, DNA extraction, fragmentation and purification. Detailed protocols are available on protocols.io ( Denton *et al.*, 2023b). The qmCerStei3 sample was prepared for DNA extraction by weighing and dissecting it on dry ice ( Jay *et al.*, 2023). Tissue from the terminal body was homogenised using a PowerMasher II tissue disruptor ( Denton *et al.*, 2023a). HMW DNA was extracted using the Automated MagAttract v2 protocol ( Oatley *et al.*, 2023a). DNA was sheared into an average fragment size of 12–20 kb in a Megaruptor 3 system ( Bates *et al.*, 2023). Sheared DNA was purified by solid-phase reversible immobilisation, using AMPure PB beads to eliminate shorter fragments and concentrate the DNA ( Oatley *et al.*, 2023b). The concentration of the sheared and purified DNA was assessed using a Nanodrop spectrophotometer and Qubit Fluorometer using the Qubit dsDNA High Sensitivity Assay kit. Fragment size distribution was evaluated by running the sample on the FemtoPulse system. For this sample, the extracted DNA had a Qubit concentration of 29.6 ng/μL and a yield of 3,848.00 ng. Spectrophotometric measurements indicated 260/280 and 260/230 ratios of 1.87 and 17.79, respectively.

RNA was extracted from posterior body tissue of qmCerStei5 in the Tree of Life Laboratory at the WSI using the RNA Extraction: Automated MagMax™ *mir*Vana protocol ( do Amaral *et al.*, 2023). The RNA concentration was assessed using a Nanodrop spectrophotometer and a Qubit Fluorometer using the Qubit RNA Broad-Range Assay kit. Analysis of the integrity of the RNA was done using the Agilent RNA 6000 Pico Kit and Eukaryotic Total RNA assay.

### Hi-C sample preparation and crosslinking

Hi-C data were generated from 20–50 mg of frozen leg tissue of the qmCerStei1 sample using the Arima-HiC v2 kit (Arima Genomics). As per manufacturer’s instructions, tissue was fixed, and the DNA crosslinked using a TC buffer with a final formaldehyde concentration of 2%. The tissue was then homogenised using the Diagnocine Power Masher-II. The crosslinked DNA was digested using a restriction enzyme master mix, then biotinylated and ligated. A clean up was performed with SPRIselect beads prior to library preparation. DNA concentration was quantified using the Qubit Fluorometer v4.0 (Thermo Fisher Scientific) and Qubit HS Assay Kit and sample biotinylation percentage was estimated using the Arima-HiC v2 QC beads.

### Library preparation and sequencing

Library preparation and sequencing were performed at the WSI Scientific Operations core.

***PacBio HiFi***

Samples with an average fragment size greater than 8 kb and total mass exceeding 400 ng were eligible for the low-input SMRTbell Prep Kit 3.0 protocol (Pacific Biosciences, California, USA), depending on genome size and required sequencing depth. Libraries were prepared using the SMRTbell Prep Kit 3.0 according to the manufacturer’s instructions. The kit includes reagents for end repair/A-tailing, adapter ligation, post-ligation SMRTbell bead clean-up, and nuclease treatment. Size selection and clean-up were performed using diluted AMPure PB beads (Pacific Biosciences). DNA concentration was quantified using a Qubit Fluorometer v4.0 (ThermoFisher Scientific) and the Qubit 1X dsDNA HS assay kit. Final library fragment size was assessed with the Agilent Femto Pulse Automated Pulsed Field CE Instrument (Agilent Technologies) using the gDNA 55 kb BAC analysis kit.

The sample was sequenced on a Revio instrument (Pacific Biosciences). The prepared library was normalised to 2 nM, and 15 μL was used for making complexes. Primers were annealed and polymerases bound to generate circularised complexes, following the manufacturer’s instructions. Complexes were purified using 1.2X SMRTbell beads, then diluted to the Revio loading concentration (200–300 pM) and spiked with a Revio sequencing internal control. The sample was sequenced on a Revio 25M SMRT cell. The SMRT Link software (Pacific Biosciences), a web-based workflow manager, was used to configure and monitor the run and to carry out primary and secondary data analysis.

***Hi-C***

For Hi-C library preparation, the biotinylated DNA constructs were fragmented using a Covaris E220 sonicator and size-selected to 400–600 bp using SPRISelect beads. DNA was then enriched using Arima-HiC v2 Enrichment beads. The NEBNext Ultra II DNA Library Prep Kit (New England Biolabs) was used for end repair, A-tailing, and adapter ligation, following a modified protocol in which library preparation is carried out while the DNA remains bound to the enrichment beads. PCR amplification was performed using KAPA HiFi HotStart mix and custom dual-indexed adapters (Integrated DNA Technologies) in a 96-well plate format. Depending on sample concentration and biotinylation percentage determined at the crosslinking stage, samples were amplified for 10–16 PCR cycles. Post-PCR clean-up was carried out using SPRISelect beads. The libraries were quantified using the Accuclear Ultra High Sensitivity dsDNA Standards Assay kit (Biotium) and normalised to 10 ng/μL before sequencing. Hi-C sequencing was performed on the Illumina NovaSeq 6000 instrument with 150 bp paired-end reads.

***RNA***

Libraries were prepared using the NEBNext ^®^ Ultra™ II Directional RNA Library Prep Kit for Illumina (New England Biolabs), following the manufacturer’s instructions. Poly(A) mRNA in the total RNA solution was isolated using oligo(dT) beads, converted to cDNA, and uniquely indexed; 14 PCR cycles were performed. Libraries were size-selected to produce fragments between 100–300 bp. Libraries were quantified, normalised, pooled to a final concentration of 2.8 nM, and diluted to 150 pM for loading. Sequencing was carried out on the Illumina NovaSeq 6000 instrument.

### Genome assembly, curation and evaluation

***Assembly***

Prior to assembly of the PacBio HiFi reads, a database of *k*-mer counts ( *k* = 31) was generated from the filtered reads using FastK. GenomeScope2 ( Ranallo-Benavidez *et al.*, 2020) was used to analyse the *k*-mer frequency distributions, providing estimates of genome size, heterozygosity, and repeat content.

The HiFi reads were first assembled using Hifiasm ( Cheng *et al.*, 2021) with the --primary option. Haplotypic duplications were identified and removed using purge_dups ( Guan *et al.*, 2020). The Hi-C reads ( Rao *et al.*, 2014) were mapped to the primary contigs using bwa-mem2 ( Vasimuddin *et al.*, 2019), and the contigs were scaffolded using YaHS ( Zhou *et al.*, 2023) using the --break option for handling potential misassemblies. The scaffolded assemblies were evaluated using Gfastats ( Formenti *et al.*, 2022), BUSCO ( Manni *et al.*, 2021) and MERQURY.FK ( Rhie *et al.*, 2020).

The mitochondrial genome was assembled using MitoHiFi ( Uliano-Silva *et al.*, 2023), which runs MitoFinder ( Allio *et al.*, 2020) and uses these annotations to select the final mitochondrial contig and to ensure the general quality of the sequence.

***Assembly curation***

The assembly was decontaminated using the Assembly Screen for Cobionts and Contaminants (ASCC) pipeline. Flat files and maps used in curation were generated via the TreeVal pipeline ( Pointon *et al.*, 2023). Manual curation was conducted primarily in PretextView ( Harry, 2022) and HiGlass ( Kerpedjiev *et al.*, 2018), with additional insights provided by JBrowse2 ( Diesh *et al.*, 2023). Scaffolds were visually inspected and corrected as described by Howe *et al*. (2021). Any identified contamination, missed joins, and mis-joins were amended, and duplicate sequences were tagged and removed. The curation process is documented at https://gitlab.com/wtsi-grit/rapid-curation.

***Assembly quality assessment***

The Merqury.FK tool ( Rhie *et al.*, 2020), run in a Singularity container ( Kurtzer *et al.*, 2017), was used to evaluate *k*-mer completeness and assembly quality for the primary and alternate haplotypes using the *k*-mer databases ( *k* = 31) computed prior to genome assembly. The analysis outputs included assembly QV scores and completeness statistics.

The blobtoolkit pipeline is a Nextflow ( Di Tommaso *et al.*, 2017) port of the previous Snakemake Blobtoolkit pipeline ( Challis *et al.*, 2020). It aligns the PacBio reads in SAMtools and minimap2 ( Li, 2018) and generates coverage tracks for regions of fixed size. In parallel, it queries the GoaT database ( Challis *et al.*, 2023) to identify all matching BUSCO lineages to run BUSCO ( Manni *et al.*, 2021). For the three domain-level BUSCO lineages, the pipeline aligns the BUSCO genes to the UniProt Reference Proteomes database ( Bateman *et al.*, 2023) with DIAMOND blastp ( Buchfink *et al.*, 2021). The genome is also divided into chunks according to the density of the BUSCO genes from the closest taxonomic lineage, and each chunk is aligned to the UniProt Reference Proteomes database using DIAMOND blastx. Genome sequences without a hit are chunked using seqtk and aligned to the NT database with blastn ( Altschul *et al.*, 1990). The blobtools suite combines all these outputs into a blobdir for visualisation.

The blobtoolkit pipeline was developed using nf-core tooling ( Ewels *et al.*, 2020) and MultiQC ( Ewels *et al.*, 2016), relying on the Conda package manager, the Bioconda initiative ( Grüning *et al.*, 2018), the Biocontainers infrastructure ( da Veiga Leprevost *et al.*, 2017), as well as the Docker ( Merkel, 2014) and Singularity ( Kurtzer *et al.*, 2017) containerisation solutions.

Table 4 contains a list of relevant software tool versions and sources.

**Table 4.** Software tools: versions and sources.

Software tool	Version	Source
BLAST	2.14.0	ftp://ftp.ncbi.nlm.nih.gov/blast/executables/blast+/
BlobToolKit	4.3.9	https://github.com/blobtoolkit/blobtoolkit
BUSCO	5.5.0	https://gitlab.com/ezlab/busco
bwa-mem2	2.2.1	https://github.com/bwa-mem2/bwa-mem2
DIAMOND	2.1.8	https://github.com/bbuchfink/diamond
fasta_windows	0.2.4	https://github.com/tolkit/fasta_windows
FastK	666652151335353eef2fcd58880bcef5bc2928e1	https://github.com/thegenemyers/FASTK
GenomeScope2.0	2.0.1	https://github.com/tbenavi1/genomescope2.0
Gfastats	1.3.6	https://github.com/vgl-hub/gfastats
GoaT CLI	0.2.5	https://github.com/genomehubs/goat-cli
Hifiasm	0.19.8-r603	https://github.com/chhylp123/hifiasm
HiGlass	44086069ee7d4d3f6f3f0012569789ec138f42b84 aa44357826c0b6753eb28de	https://github.com/higlass/higlass
MerquryFK	d00d98157618f4e8d1a9190026b19b471055b22e	https://github.com/thegenemyers/MERQURY.FK
Minimap2	2.24-r1122	https://github.com/lh3/minimap2
MitoHiFi	2	https://github.com/marcelauliano/MitoHiFi
MultiQC	1.14, 1.17, and 1.18	https://github.com/MultiQC/MultiQC
Nextflow	23.04.1	https://github.com/nextflow-io/nextflow
PretextView	0.2.5	https://github.com/sanger-tol/PretextView
purge_dups	1.2.3	https://github.com/dfguan/purge_dups
samtools	1.19.2	https://github.com/samtools/samtools
sanger-tol/ascc	0.1.0	https://github.com/sanger-tol/ascc
sanger-tol/blobtoolkit	0.4.0	https://github.com/sanger-tol/blobtoolkit
Seqtk	1.3	https://github.com/lh3/seqtk
Singularity	3.9.0	https://github.com/sylabs/singularity
TreeVal	1.2.0	https://github.com/sanger-tol/treeval
YaHS	1.1a.2	https://github.com/c-zhou/yahs

### Wellcome Sanger Institute – Legal and Governance

The materials that have contributed to this genome note have been supplied by a Darwin Tree of Life Partner. The submission of materials by a Darwin Tree of Life Partner is subject to the **‘Darwin Tree of Life Project Sampling Code of Practice’**, which can be found in full on the Darwin Tree of Life website here. By agreeing with and signing up to the Sampling Code of Practice, the Darwin Tree of Life Partner agrees they will meet the legal and ethical requirements and standards set out within this document in respect of all samples acquired for, and supplied to, the Darwin Tree of Life Project. 

Further, the Wellcome Sanger Institute employs a process whereby due diligence is carried out proportionate to the nature of the materials themselves, and the circumstances under which they have been/are to be collected and provided for use. The purpose of this is to address and mitigate any potential legal and/or ethical implications of receipt and use of the materials as part of the research project, and to ensure that in doing so we align with best practice wherever possible. The overarching areas of consideration are:

•     Ethical review of provenance and sourcing of the material

•     Legality of collection, transfer and use (national and international)

Each transfer of samples is further undertaken according to a Research Collaboration Agreement or Material Transfer Agreement entered into by the Darwin Tree of Life Partner, Genome Research Limited (operating as the Wellcome Sanger Institute), and in some circumstances other Darwin Tree of Life collaborators.

## Data Availability

European Nucleotide Archive: Ceratothoa steindachneri. Accession number PRJEB72113; https://identifiers.org/ena.embl/PRJEB72113. The genome sequence is released openly for reuse. The *Ceratothoa steindachneri* genome sequencing initiative is part of the Darwin Tree of Life (DToL) project. All raw sequence data and the assembly have been deposited in INSDC databases. Raw data and assembly accession identifiers are reported in Table 1 and Table 2.

## Author information

Members of the Natural History Museum Genome Acquisition Lab are listed here: https://doi.org/10.5281/zenodo.12159242.

Members of the Darwin Tree of Life Barcoding collective are listed here: https://doi.org/10.5281/zenodo.12158331.

Members of the Wellcome Sanger Institute Tree of Life Management, Samples and Laboratory team are listed here: https://doi.org/10.5281/zenodo.12162482.

Members of Wellcome Sanger Institute Scientific Operations: Sequencing Operations are listed here: https://doi.org/10.5281/zenodo.12165051.

Members of the Wellcome Sanger Institute Tree of Life Core Informatics team are listed here: https://doi.org/10.5281/zenodo.12160324.

Members of the Tree of Life Core Informatics collective are listed here: https://doi.org/10.5281/zenodo.12205391.

Members of the Darwin Tree of Life Consortium are listed here: https://doi.org/10.5281/zenodo.4783558.
