# Supplementary material for: The genome sequence of the tongue-biting isopod, Ceratothoa steindachneri Koelbel, 1878
Source: Wellcome Open Res. 2025 May 27;10:278. [Version 1] doi: 10.12688/wellcomeopenres.24289.1 (PMC12361884; doi:10.12688/wellcomeopenres.24289.1)
Supplement: Peer review history

# Reviewer report: Reviewer response for version 1

**DOI:** 10.21956/wellcomeopenres.26787.r125846

**Published:** 2025-08-18

**Authors:** Christopher J. Fields

**Affiliations:** 1 Carver Biotechnology Center, University of Illinois Urbana-Champaign, Urbana, 61801, USA

The submission describes the genome assembly of the tongue-biting isopod (*Ceratothoa steindachneri*), a keg of parasitic nightmare fuel. The overall paper describes the general process and steps taken for sample collection, library preparation, and assembly, with reasonable descriptions for each step including some of the manual steps in curation during the assembly process.  However, a few points should be addressed for better data reproduction.

 **Primary concerns:** I do have a few questions about the assembly steps which would be great to clarify.

 1) `purge_dups` is mentioned as being used for removing redundancies in the assembly, I’m assuming since this is effectively a haploid assembly based on the sample being sequenced and the use of `--primary` in the `hifiasm` assembly step.  How was this particular step run?

 2) `Merqury.FK` is mentioned as being used in QA/QC, but no steps are described on how this was performed:

 > The Merqury.FK tool (~Rhie et al., 2020~), run in a Singularity container (~Kurtzer et al., 2017~), was used to evaluate *k*-mer completeness and assembly quality for the primary and alternate haplotypes using the *k*-mer databases (*k* = 31) computed prior to genome assembly. The analysis outputs included assembly QV scores and completeness statistics.

 3) Though the tools are outlined with versions, several workflows are also mentioned which do not have versions or tags/hashs for the versions used, and (in cases where no reference is available) do not have links to the relevant code; it would be great to at least have links.  For example:

 > * ‘The assembly was decontaminated using the Assembly Screen for Cobionts and Contaminants (ASCC) pipeline…’

 > ‘Flat files and maps used in curation were generated via the TreeVal pipeline (~Pointon et al., 2023~).’

 > * ‘The blobtoolkit pipeline is a Nextflow (~Di Tommaso et al., 2017~) port of the previous Snakemake Blobtoolkit pipeline (~Challis et al., 2020~).’

 4) Annotation is mentioned as being performed externally using the Ensembl annotation pipeline, but there isn’t a reference given for this (I’m assuming this is genebuild?).  Inputs for the annotation are also not mentioned; I’m assuming the RNA-Seq data was utilized, but this isn’t indicated.

Are sufficient details of methods and materials provided to allow replication by others?

Partly

Is the rationale for creating the dataset(s) clearly described?

Yes

Are the datasets clearly presented in a useable and accessible format?

Partly

Are the protocols appropriate and is the work technically sound?

Yes

Reviewer Expertise:

Computational Genomics

I confirm that I have read this submission and believe that I have an appropriate level of expertise to confirm that it is of an acceptable scientific standard, however I have significant reservations, as outlined above.

# Reviewer report: Reviewer response for version 1

**DOI:** 10.21956/wellcomeopenres.26787.r125843

**Published:** 2025-07-28

**Authors:** Michael Dondrup

**Affiliations:** 1 Department of Informatics, University of Bergen, Thormøhlensgate, Norway

The authors describe the genome assembly of the parasitic isopod *Ceratothoa steindachneri, *representing **an organism with a unique lifestyle from an undersampled group.  

 The methods, assembly, and quality metrics are presented clearly in a standardized manner, accompanied by specimen photographs. The Ensembl annotation further adds to the value of this genome sequence.

Are sufficient details of methods and materials provided to allow replication by others?

Yes

Is the rationale for creating the dataset(s) clearly described?

Yes

Are the datasets clearly presented in a useable and accessible format?

Yes

Are the protocols appropriate and is the work technically sound?

Yes

Reviewer Expertise:

Bionformatics, genomics, transcriptomics, data brokering (I am currently involved in data-brokering to ENA for EBP Norway)

I confirm that I have read this submission and believe that I have an appropriate level of expertise to confirm that it is of an acceptable scientific standard.

# Reviewer report: Reviewer response for version 1

**DOI:** 10.21956/wellcomeopenres.26787.r125194

**Published:** 2025-06-24

**Authors:** Stephan Koblmüller

**Affiliations:** 1 University of Graz, Graz, Graz, Austria

In this manuscript, Fletcher et al. present the whole genome assembly for the isopod *Ceratothoa steindachneri*, an ectoparasite of marine fish. Typically, some background information is given on the sequenced species, as well as a rationale for sequencing it. The general background on the species is given here, but a rationale for sequencing the whole genome of this species is largely lacking (-> why was the species sequenced? Any particular reason for why this particular species was chosen? ...). I suggest adding a few words (1-2 sentences) to the intro.

 The data are clear; the methods are well described. I have no objections to any of the data presented or the results. As far as I can tell, the datasets are deposited in / accessible from publicly accessible repositories.

Are sufficient details of methods and materials provided to allow replication by others?

Yes

Is the rationale for creating the dataset(s) clearly described?

Partly

Are the datasets clearly presented in a useable and accessible format?

Yes

Are the protocols appropriate and is the work technically sound?

Yes

Reviewer Expertise:

phylogenetics, phylogenomics, population genetics, phylogeography

I confirm that I have read this submission and believe that I have an appropriate level of expertise to confirm that it is of an acceptable scientific standard, however I have significant reservations, as outlined above.
